# How does a surgeon’s brain buzz? An EEG coherence study on the interaction between humans and robot

**DOI:** 10.1186/1744-9081-9-14

**Published:** 2013-04-22

**Authors:** Tommaso Bocci, Carlo Moretto, Silvia Tognazzi, Lucia Briscese, Megi Naraci, Letizia Leocani, Franco Mosca, Mauro Ferrari, Ferdinando Sartucci

**Affiliations:** 1 Department of Neuroscience, Unit of Neurology, Pisa University Medical School, Pisa, Italy; 2 Department of Neurological and Neurosensorial Sciences, Neurology and Clinical Neurophysiology Section, Azienda Ospedaliera Universitaria Senese, Siena, Italy; 3 Bariatric and Metabolic Surgical Unit, Azienda Ospedaliero-Universitaria Pisana, Pisa, Italy; 4 Department of Neuroscience, Cisanello Neurology Unit, Azienda Ospedaliera Universitaria Pisana, Pisa, Italy; 5 Institute of Experimental Neurology, Division of Neuroscience, Scientific Institute and University Ospedale San Raffaele, Milan, Italy; 6 Division of General Surgery, Pisa University Medical School, Pisa, Italy; 7 Department of Vascular Surgery, Pisa University Medical School, Pisa, Italy; 8 CNR Neuroscience Institute, Pisa, Italy; 9 Neuroscience Department, Neurology - Neurophysiology Units, Pisa University Medical School, Via Roma, n. 67, Pisa, I 56126, Italy

**Keywords:** Robotic surgery, Laparoscopy, Da Vinci, EEG coherence, Interhemispheric coherence, M1, S1, SMA, Pre-SMA, Mirror neurons

## Abstract

**Introduction:**

In humans, both primary and non-primary motor areas are involved in the control of voluntary movements. However, the dynamics of functional coupling among different motor areas have not been fully clarified yet. There is to date no research looking to the functional dynamics in the brain of surgeons working in laparoscopy compared with those trained and working in robotic surgery.

**Experimental procedures:**

We enrolled 16 right-handed trained surgeons and assessed changes in intra- and inter-hemispheric EEG coherence with a 32-channels device during the same motor task with either a robotic or a laparoscopic approach. Estimates of auto and coherence spectra were calculated by a fast Fourier transform algorithm implemented on Matlab 5.3.

**Results:**

We found increase of coherence in surgeons performing laparoscopy, especially in theta and lower alpha activity, in all experimental conditions (M1 vs. SMA, S1 vs. SMA, S1 vs. pre-SMA and M1 vs. S1; p < 0.001). Conversely, an increase in inter-hemispheric coherence in upper alpha and beta band was found in surgeons using the robotic procedure (right vs. left M1, right vs. left S1, right pre-SMA vs. left M1, left pre-SMA vs. right M1; p < 0.001).

**Discussion:**

Our data provide a semi-quantitative evaluation of dynamics in functional coupling among different cortical areas in skilled surgeons performing laparoscopy or robotic surgery. These results suggest that motor and non-motor areas are differently activated and coordinated in surgeons performing the same task with different approaches. To the best of our knowledge, this is the first study that tried to assess semi-quantitative differences during the interaction between normal human brain and robotic devices.

## Introduction

In the human brain, motor and non-motor areas are functionally bound together to work as a global network both in planning and performing a motor act. Our survival and success in everyday life depend on our ability to coordinate and integrate several different tasks [1-3]. The degree of connectivity between spatially distinct pairs of brain sources may be evaluated by coherence, a statistical estimate of the correlation coefficient between two time series in the same frequency [4,5]. High coherence likely reflects substantial temporal coordination in the electrocortical activity between two locations, whereas low values suggest electrocortical activity in each location is independent and autonomous [6,7]. For instance, functional neuroimaging studies have revealed a great activation of premotor areas in patients with ischemic lesions in the primary motor cortex, M1 [8-10]; in this view, the functional coupling among different cortical areas could represent the physiological substrate of large scale motor plasticity, particularly in subjects with regional dysfunctions [11]. Besides, EEG coherence is a mark of physiological brain development and maturation [12-14] increasing with conceptional and chronological age especially in delta and beta range and in a non-linear fashion [15-17]. Finally, the synchronization of cortical activity between the two hemispheres has been proposed to represent the natural neural substrate for Gestalt-type perceptual operations [18].

Study of interhemispheric coherence allows us to evaluate callosal contribution to cortical activity driving; research in animal models showed that coherence mediated by cortico–cortical connections predominates over thalamo–cortical one within the α-range, implying that cortico–cortical connectivity is the main substrate for the α-rhythm synchronization [19,20]: thus, EEG interhemispheric coherence can be used to study specific neurological or psychiatric diseases with abnormalities in cortico-cortical connectivity [21].

In spite of a wide literature describing coherence modifications in several pathological conditions [12,22-27], there is a substantial lack of studies evaluating coherence patterns in healthy humans performing different motor tasks or the same task with different approaches.

To the best of our knowledge, there is to date no research looking to the functional changes in the normal human brain during the interaction with a robotic device. Clinically, that could be of particular interest in the evaluation of dynamic changes in the brain of patients wearing artificial limbs or undergoing robot-aided rehabilitation programs [28,29]. Moreover, the growing use of brain computer interface (BCI) devices allows many patients to translate brain signals or ocular movements into commands, overtaking dramatic motor difficulties present in motorneurons disorders or other neurological syndromes [30].

In the present study, we evaluated brain functional changes under a particular experimental condition. To date, the choice between laparoscopy and robotic surgery has been made on the basis of the surgeon’s experience only: there is no evidence in literature that robotic procedures are better than laparoscopy or open surgery and no randomized trial regarding this issue has been reported yet. Today, robotic procedures are extensively used in a wide number of surgical applications, ranging from the management of gynecologic malignancies [31-33] to resection of urinary [34-36] and digestive tract tumors [37,38].

Many surgeons say that robotic procedures are easier and more effective for the patient [39]. For this reason we have aimed to investigate the influence of robot on brain dynamics of the operator. In this study, we enrolled 16 right-handed surgeons, with the same experience in the field of robotic and laparoscopic surgery; we evaluated modifications in intra- and inter-hemispheric EEG coherence with a 32-channels device during the same motor task with either a robotic or a laparoscopic approach.

## Materials and methods

### Subjects

We enrolled 16 surgeons (6 female and 10 males; mean age ± SD: 37 ± 4.1 years), all with normal or corrected-to-normal vision and no history of neurological or psychiatric disorders. They were all right-handed and had the same experience in the field of laparoscopic and robotic surgery (7.3 ± 2.2 and 7.1 ± 2.8 years, respectively).

No subject was taking any medication at the time of, or one month before, the inclusion in the study and they all had suspended alcohol consumption at least 48 hours before. To check the within-subject reliability of our data, the EEGs of four subjects were recorded twice during time-separated different sessions. Written informed consent was obtained from all subjects prior to the inclusion in the study that had been previously approved by the Local Ethical Committee and followed the tenets of Helsinki declaration.

### Motor task and robot workspace

The same motor task was performed by 16 surgeons using a laparoscopic and a robotic approach and the sequence of those surgical tasks were randomized. Each surgeon had to place three stitches in three different black targets designed on a gel case; the surgical motion should be performed bimanually, with the same sequence and following the basic surgical rules as regards placing stitches. By laparoscopy, the surgeon needs to operate watching a monitor (with a bi-dimensional view) using an optic and straight instruments while in robotic surgery, using the da Vinci® Surgical System (Intuitive Surgical Inc., Sunnyvale, CA), for which the vision is tridimensional and the surgical instruments are articulated on their end like a wrist. Those features give the surgeon more dexterity during complex surgical laparoscopic procedure, like suturing or isolating delicate anatomical structures, and it is probably less stressful.

A laparoscopic Karl Storz cart was used to perform the laparoscopic surgical skills: the surgeon operated watching a monitor with a bi-dimensional view using a Karl Storz 10 mm 30° optic with a straight laparoscopic needle driver in the right hand and a straight cadiere forcep in the left one. Each exercise was located into a pelvic trainer (Ethicon Endosurgery) in order to simulate an abdominal working space.

The teleoperated robotic system is based on a master–slave control concept. It consists of two major units. The surgeon’s console unit houses the display system, the surgeon’s user interface and the electronic controller. The second unit consists of four manipulators, three for telemanipulation of surgical instruments and one dedicated to the endoscopic camera [40]. The system restores degrees of freedom (DOFs) lost in conventional laparoscopy; the three-DOF wrist inside the patient allows natural wrist pronation/supination, providing a total of seven DOFs for instrument tip control (three orientations, three translations and grip).

The thumb and index finger of each hand are placed in a gripper interface, attached to each handle of the distal part of the master interface, by means of adjustable Velcro straps. The surgeon’s fingers are virtually connected to the jaws of the instrument tip. Each handle allows rotations around the three Cartesian axes of a reference frame fixed on the handle.

Despite complexity, the motor task performed in robotic surgery allows to reduce variability in the evaluation of EEG coherence due to different movements and duration of epochs among subjects and between the two experimental conditions (laparoscopic and robotic). First, a remote centre of motion (RCM) creates a fulcrum point distally located from the structure itself: that allows a correct orientation of the robotic instrument without changing position of the entry point. Second, the console provides an immersive stereoscopic viewing. Third, predominant bimanual movements in robotic surgery eliminates both hand tremor and innate handedness [41,42].

During the surgical performance an EEG was recorded and collected and all the recordings were made with the same EEG montage.

### EEG data collection and processing

EEG data were continuously recorded by means of a 32-channel Geodesic Sensor Net (Electrical Geodesics Inc., EGI, Eugene, Oregon) at rest (baseline) and during the execution of the same motor task, in both laparoscopy and robotic surgery.

At rest, during the EEG recording sessions, the subjects were sitting in a comfortable armchair in a silent room in which the temperature was kept constant; they were all at full psychosensorial rest, both with closed and open eyes. On the other hand, EEG was continuously recorded during the execution of the entire task, with time series having different duration among subjects and between the two experimental conditions; thus, for coherence analysis we used time series with different lengths. We did not include epochs before or after the movements.

During acquisition, impedance was kept below 5 kΩ. Electrooculogram (EOG) was recorded simultaneously with a bipolar electrode to remove EEG artifacts induced by eye movements. The common reference (Cz) has been widely used in studies of EEG coherence [43]. EEG signals were sampled at 250 Hz, digitized with a 16-bit analog-to-digital converter (ADC), and filtered using an analog filter from 1 to 50 Hz. The data were then resampled at 256 sample/s in Brain Vision Analyzer (version 1.05, Brain Products, GmbH, Gilching, Germany), to allow selection of appropriate frequency bands for the calculation of coherence. The data were edited off-line for ocular and movement artifact, using a ±100 μV criterion, in Brain Vision Analyzer. If artifacts were detected in one channel, data from all remaining channels were excluded for that specific epoch.

Because coherence data depend on the recording type [44] and, in particular, are highly sensitive to signal variations at the common reference [45], we further checked the intra-individual reproducibility of our results under different montage schemes. We off-line converted our initial Cz reference into common average, bipolar, and ipsilateral earlobe reference montages, which all emphasize different properties of the EEG signals. Under these experimental conditions, and with the aid of a high density recording system, any observed stimulus – dependent change in interhemispheric coherence cannot be ascribed to the influence of a common reference, each hemisphere having its own independent reference electrode [46,47]. Another problem for a high-density EEG is about spurious coherence due to volume conduction effects: however, since we were mainly interested in comparing coherence between contrasting surgical approaches, rather than assessing contribution of different cortical areas, concerns about volume conduction are of secondary relevance [33].

Power spectrum density (PSD) for every channel and coherence functions for interhemispheric pairs of leads were calculated by averaging the primary spectral estimates (computed by fast Fourier transformation) over all epochs and smoothing the averages by Parzen’s window.

### Cortical functional mapping

In this study, we considered five areas of interest in both hemispheres: primary motor area (M1), primary somatosensory area (S1), primary visual area (V1), supplementary motor area (SMA) and pre-supplementary motor area (pre-SMA).

Intra-hemispheric EEG coherence was bilaterally evaluated between the following areas: M1 vs S1 (C3-Cz vs P3-Pz and C4-Cz vs P4-Pz), M1 vs SMA (C3-Cz vs FC3-Fz and C4-Cz vs FC4-Fz), S1 vs SMA (P3-Pz vs FC3-Fz and P4-Pz vs FC4-Fz), M1 vs pre-SMA (C3-Cz vs FC5-Fz and C4-Cz vs FC6-Fz) and S1 vs pre-SMA (P3-Pz vs FC5-Fz and P4-Pz vs FC6-Fz). Inter-hemispheric EEG coherence was measured between the following 4 areas: left vs right M1 (C3-C4), left vs right S1 (P3-P4), left M1 vs right pre-SMA (C3-Cz vs FC6-Fz) and right M1 vs left pre-SMA (C4-Cz vs FC5-Fz); Global relative EEG powers pooled from recording channels were computed in 3.5-19.5 Hz range with a resolution of 0.98 Hz. We didn’t compute delta band (< 3.5 Hz) for its very negligible amount in all subjects.

Anatomical location of the VAC (vertical through the center of the anterior commissure) line for the determination of pre-supplementary motor area was determined based on a study-specific template obtained from T1-weighted MRI images of a sample of six surgeons.

### Power spectrum and coherence analysis

As a preliminary step, the spectral properties of the EEG signals recorded were investigated. In particular, the power band ratios of the EEG recordings were estimated for the laparoscopic and robotic condition. These quantities are defined as follows: *R*_*theta*_ = *P*_*theta*_/*P*_*Tot*_, *R*_*alpha*-*1*_ = *P*_*alpha*-*1*_/*P*_*tot*_, *R*_*alpha*-*2*_ = *P*_*alpha*-*2*_/*P*_*tot*_ and *R*_*beta*_ = *P*_*beta*_/*P*_*Tot*_ where *P*_*Tot*_ is the total power, while *P*_*X*_ (*X* = *Theta*, *Alpha*-*1*, *Alpha*-*2 and Beta*) is the power in the corresponding band (theta, 3.5–8.0 Hz; alpha-1 band, 8.0–10.5 Hz; alpha-2 band, 10.5–13.0 Hz; and beta band, 13–19.5 Hz).

Power spectrum density (PSD) for every channel and coherence functions (Coh) were calculated by averaging the primary spectral estimates (computed by fast Fourier transformation) over all epochs and smoothing the averages by Parzen’s window. Coherence function between two signals *x* and *y* at each frequency *f* was then calculated as:

(1) $$\mathrm{Co}h_{\mathrm{xy}}\left(f\right)={\left|S_{\mathrm{xy}}\left(f\right)\right|/\left[S_{\mathrm{xx}}\left(f\right)*S_{\mathrm{yy}}\left(f\right)\right]}^{1/2}$$

where *S*_*xy*_( *f* ) is the cross power spectral density, which is the distribution of power per unit frequency, while *S*_*xx*_( *f* ) and *S*_*yy*_( *f* ) are power spectral densities of the *x* and *y* signals. Over successive epochs of analysis, the coherence estimate depends upon power and phase dynamics of the two signals and reflects strength of linear relationship between two channels. Its value ranges between 0 (the two signals are linearly independent) and 1 (linear relationship between the two signals).

### Statistical analysis

Estimates of auto and coherence spectra were calculated by a fast Fourier transform algorithm implemented on Matlab 5.3 (Mathworks, Natick, MA).

A two-way analysis of variance (ANOVA), followed by Duncan post-hoc test, was used for statistical purposes. Power spectrum was analyzed separately for each frequency band in mixed model ANOVAs with group (laparoscopy, robotic), frequencies and regions (M1, S1, SMA, pre-SMA, V1) as factors. Differences in EEG coherence were assessed similarly, in a two-way factorial ANOVA with group (laparoscopy, robotic), sex, hemisphere (left, right) and regions (M1, S1, SMA, pre-SMA, V1) as factors. A one-way ANOVA was used to compare duration of the motor task, as an indirect measure of surgical performance, between the two experimental conditions.

To compare data collected from each subject we used the Pearson’s correlation coefficient.

## Results

### Intra-hemispheric coherence

**Figure 1:**
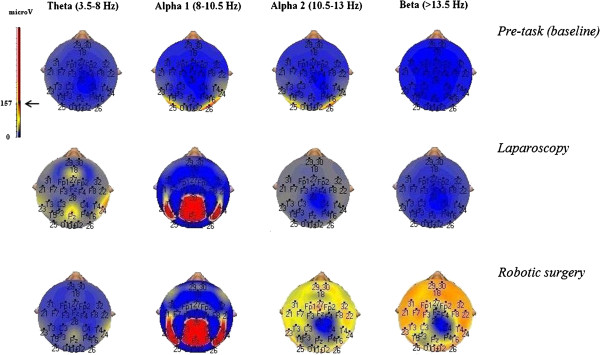
**Power spectrum maps of the same surgeon in a resting condition (pre-task, top row) and performing the motor task with either a conventional laparoscopic (middle row) or a robotic (bottom row) modality.** Note the larger representation of upper alpha and beta frequencies when a robotic approach was used.

**Figure 2:**
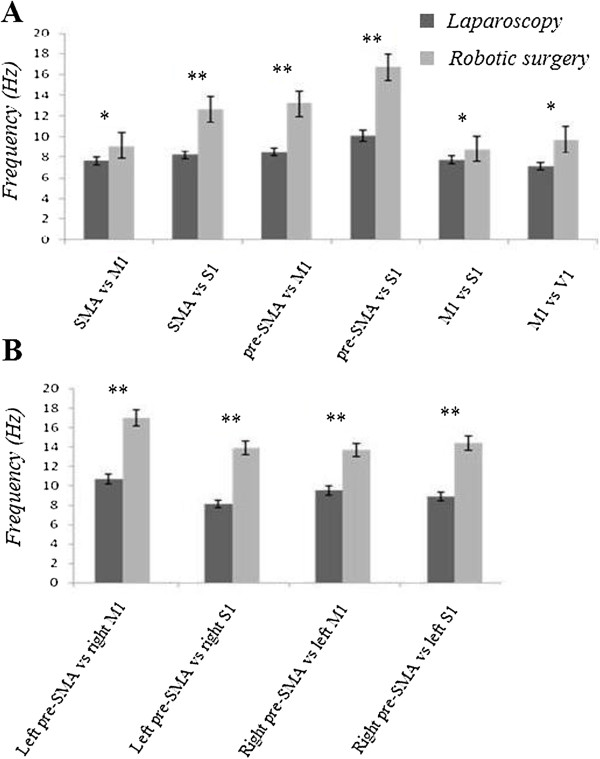
**Mean EEG frequency values for all the considered recording locations, both for intra- (A) and inter-hemispheric (B) comparisons: note that when a robotic modality was used the beta and upper alpha frequencies were the more represented.** Overall, the findings prove a different EEG driving between the two operating modalities in spectral frequency, as concerns intra- as well as inter-hemispheric analysis.

Figure 1 shows power spectrum maps for theta, lower alpha, upper alpha and beta activity in a surgeon at rest (baseline, top row) or performing the same task with either a laparoscopic (middle maps) or a robotic (bottom maps) modality. Figure 2 shows mean frequency values in EEG recordings for all the comparisons made.

**Figure 3:**
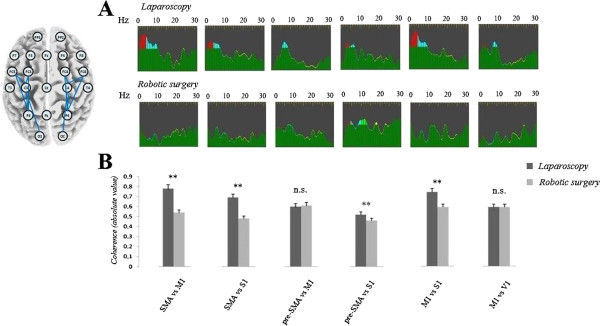
**Intra-hemispheric coherence changes between laparoscopic and robotic modality.** Sample of intra-hemispheric coherence changes in the same surgeon operating with a laparoscopic (top traces) or a robotic (bottom traces) approach is shown in top panel (**A**). The colors represent the four frequency bands analyzed (red: delta; blue: theta; green: alpha 1 and alpha 2; yellow: beta) and were marked when the absolute value of the coherence function was > 0.5. Note the higher functional coupling, especially at theta band, in surgeons using a laparoscopic approach. The bottom panel (**B**) shows the mean absolute value of coherence function in theta and lower alpha band for all the comparisons analyzed; surgeons operating with a conventional laparoscopic procedure show significant higher values, except for the comparisons pre-SMA vs. M1 and M1 vs. V1 (**p < 0.001; *p < 0.01; n.s.: not significant). Inset on the upper left side represents an exemplificative pattern of intra-hemispheric channel comparisons.

Differences in intra-hemispheric coherence values between laparoscopic and robotic surgery are provided in Table 1 and in Figure 3.

**Table 1:** Frequency (expressed in Hz) of the first and second coherence peak (± 1 SD) both for inter- (A) and intra-hemispheric (B) comparisons

***A*.**	**BASELINE**		**LAPAROSCOPY**		**ROBOTIC SURGERY**	
	1° PEAK	2° PEAK	1° PEAK	2° PEAK	1° PEAK	2° PEAK
Frequency (Hz)						
SMA *versus*:						
M1	8.4 ± 0.5	7.8 ± 0.6	7.2 ± 0.5	7.5 ± 0.4	9.3 ± 1.0	8.5 ± 0.7
S1	7.9 ± 0.6	8.0 ± 0.7	8.0 ± 0.1	7.3 ± 0.3	12.3 ± 1.2	13.1 ± 1.4
Pre-SMA *versus*:						
M1	8.4 ± 0.4	8.7 ± 0.7	7.6 ± 0.3	7.9 ± 0.2	13.5 ± 1.8	12.6 ± 1.3
S1	9.1 ± 0.3	8.9 ± 0.5	9.5 ± 0.1	7.9 ± 0.3	15.8 ± 2.0	14.6 ± 1.4
M1 *versus* S1	8.5 ± 0.8	8.0 ± 0.6	7.9 ± 0.2	7.5 ± 0.4	8.9 ± 1.0	8.2 ± 0.8
M1 *versus* V1	9.8 ± 0.9	8.4 ± 0.6	7.2 ± 0.4	7.8 ± 0.3	8.4 ± 0.5	8.7 ± 0.6
***B*.**			1° PEAK		2° PEAK	
pre-SMA *versus* contralateral M1:						
Baseline			9.3 ± 0.5		9.4 ± 0.4	
Laparoscopy			9.0 ± 0.3		9.5 ± 0.1	
Robotic surgery			14.5 ± 1.3		15.9 ± 1.8	
pre-SMA *versus* contralateral S1:						
Baseline			7.7 ± 0.5		8.9 ± 0.8	
Laparoscopy			7.0 ± 0.2		8.1 ± 0.3	
Robotic surgery			14.1 ± 1.0		14.5 ± 1.3	

As concerns interhemispheric recordings, here we reported only the comparisons between pre-SMA and contralateral M1 or S1 area. Note the predominance of upper alpha and beta frequency during the execution of the motor task with the robotic device.

The analysis of coherence revealed a significant increase in intra-hemispheric coherence in the range of theta activity in surgeons using a conventional laparoscopic approach, compared both with resting condition (SMA vs. M1: p < 0.001; SMA vs. S1: p = 0.016; M1 vs. S1: p = 0.0024) and robotic surgery (SMA vs. M1: F_(2, 14)_ = 29.3, p < 0.001; SMA vs. S1: F_(2, 14)_ = 18.8, p < 0.001; M1 vs. S1: F_(2, 14)_ = 37.8, p < 0.001). Similar results were found by comparing S1 with pre-SMA (laparoscopy vs. resting condition: p = 0.0028; laparoscopy vs. robotic surgery: p < 0.001). As regards the lower alpha band, we showed a significant increase of coherence value in surgeons operating by laparoscopy compared with robotic surgery, for all the comparisons, although sometimes less robust than those observed for theta activity (SMA vs. M1: F_(2, 14)_ = 7.5, p < 0.05; SMA vs. S1: F_(2, 14)_ = 11.7, p < 0.01; M1 vs. S1: F_(2, 14)_ = 15.5, p < 0.001). We did not find any significant differences between males and females performing the same task (p > 0.1 for all the comparisons, both during laparoscopic and robotic procedures) neither between right and left hemispheres (p > 0.1).

### Inter-hemispheric coherence

**Figure 4:**
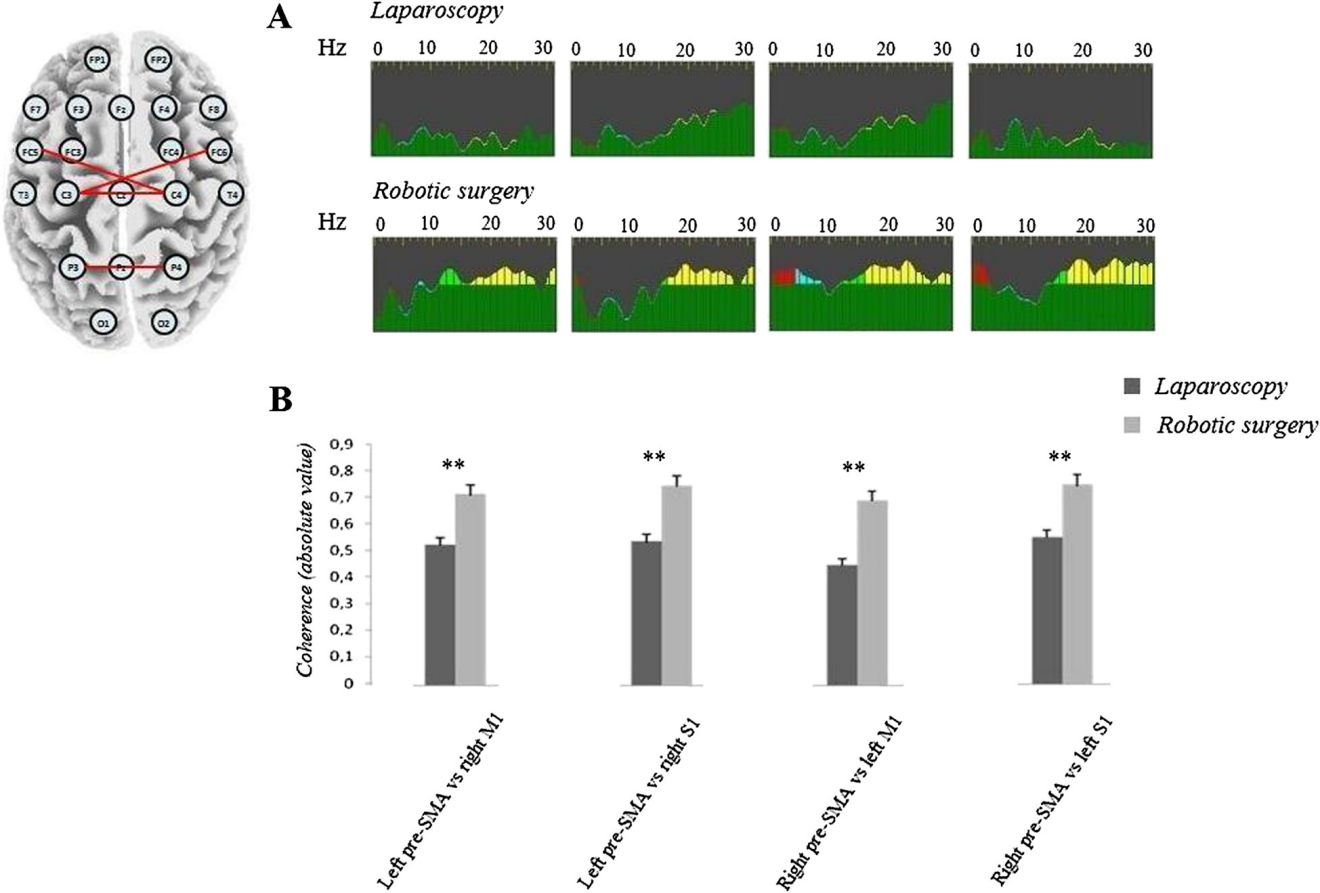
**Inter-hemispheric coherence changes between laparoscopic and robotic modality.** Sample of inter-hemispheric coherence changes in the same surgeon operating with a laparoscopic (top raw) or a robotic (bottom raw) approach is shown in panel **A**. The bottom panel (**B**) shows the mean value of coherence function in the range of upper alpha and beta band; different from intra-hemispheric coherence, surgeons operating with the robotic device show significant higher values. Inset on the upper left side represents an exemplificative pattern of inter-hemispheric channel comparisons.

Differences in inter-hemispheric coherence values between laparoscopic and robotic surgery are provided in Figure 4 and a sample of changes in the same surgeons is shown in Figure 4A.

The analysis of coherence revealed a significant increase in inter-hemispheric coherence in the range of beta activity in surgeons using the robotic device compared both with resting condition (right vs. left M1: F_(2, 14)_ = 13.7, p < 0.001; right vs. left S1: F_(2, 14)_ = 12.7, p < 0.005) and laparoscopy modality (right vs. left M1: F_(2, 14)_ = 43.8, p < 0.001; right vs. left S1: F_(2, 14)_ = 21.4, p < 0.001). Similar results were found by comparing right M1 with left pre-SMA (laparoscopy vs. resting condition: F_(2, 14)_ = 37.3, p < 0.001; laparoscopy vs. robotic surgery: F_(2, 14)_ = 23.5, p < 0.001) and left M1 with right pre-SMA (laparoscopy vs. resting condition: p < 0.001; laparoscopy vs. robotic surgery: p < 0.001). As concerns upper alpha band too, we undisclosed a significant increase of coherence value in surgeons operating with the robot compared with laparoscopy, for all the comparisons although sometimes less robust than those observed for beta activity (right vs. left M1: p < 0.001; right vs. left S1: p < 0.01; right M1 vs. left pre-SMA: p < 0.01; left M1 vs. right pre-SMA: p < 0.05). No significant difference was found in theta and lower alpha band (p > 0.1, for all the comparisons).

As reported for intra-hemispheric measures, we did not found any significant difference between males and females performing the same task (p > 0.1 for all the comparisons, both during laparoscopic and robotic procedures) neither between right and left hemispheres (p > 0.1).

Interestingly, analysis of the data from individual subjects showed that there was a significant correlation in coherence values between laparoscopic and robotic procedure. Subjects who had the largest increase in intra-hemispheric coherence during laparoscopy showed the greatest potentiation of inter-hemispheric coherence during robotic surgery (Pearson’s correlation coefficient for all the comparisons: *p* < 0.005).

### Correlation between coherence data and surgical performance

Experiments in laparoscopy had a longer duration compared with those performed with the robot (6.35’ ± 1.08’ vs. 3.27’ ± 57”; one-way ANOVA, p < 0.001). Particularly, there was a significant correlation between duration of motor task and mean intra-hemispheric coherence value in theta range during laparoscopy (one-way ANOVA: F_(1,15)_ = 9.24, p = 0.0083); concurrently, we found a significant inverse correlation between duration of motor task and mean inter-hemispheric coherence value both in alpha-2 (F_(1,15)_ = 48.1, p < 0.0001) and beta range (F_(1,15)_ = 224.1, p < 0.00001). Surgeons who had the largest increase in theta inter-hemispheric coherence during laparoscopic procedure performed more slowly the motor task (Pearson’s correlation coefficient: 0.83, with *p* < 0.0001). Similarly, surgeons who performed more rapidly the motor task in robotic procedure showed the largest increase as concerns intra-hemispheric coherence both in alpha-2 and beta range (Pearson’s correlation coefficient: - 0.94 and – 0.79, respectively, with *p* < 0.0001)

## Discussion

### Differences in power spectrum and intra-hemispheric coherence

To our knowledge, this is the first paper that tried to assess differences both in intra- and inter-hemispheric connectivity in surgeons operating in laparoscopy compared with those trained in robotic surgery. Our data provide a semi-quantitative evaluation of dynamics in functional coupling among different cortical areas in surgeons performing the same motor task with two different approaches. Power spectrum analysis confirms that the spatial distribution of coherence and power was not significantly correlated, strengthening the hypothesis that the movement-related coherence analysis specifically detects the functional linkage between motor areas, independent of the activation of each area measured by the power change [48].

We detected a wider activation of motor and non-motor areas in volunteers using a laparoscopic approach compared with those operating with the robot. In particular, we showed highly significant differences in terms of mean intra-hemispheric EEG coherence by analyzing the comparisons M1 vs SMA, S1 vs SMA, S1 vs pre-SMA and M1 vs S1; that could suggest a wider and probably more expansive activation of primary and high-order areas in surgeons using a traditional approach in the operative theater. Classically, synchronization of cortical activity is considered a marker of cognitive inactivity, active inhibition of sensory information, or silencing of unnecessary cortical areas [49,50]; however, since the surgeons made the same complex motor task, we rather agree with Knyazev and colleagues, who suggest that synchronization in alpha power improves readiness of alpha system to sensory information processing [51-53].

Consistent with this idea, power spectrum analysis revealed a more robust alpha and beta activity in surgeons using a robotic device, both in intra and inter-hemispheric comparisons (Figure 2); our hypothesis is in line with recent observations suggesting that functional interactions in the alpha and beta frequency bands are critical for better visual perception, attention and working memory functions [54-60]. In this connection, the strength of oscillatory synchrony in beta range (14–30 Hz) seems to be the more relevant parameter of the neural population dynamics that matches behavioral performance [61]. This interpretation is in keeping with findings obtained in humans showing that beta oscillatory synchrony between extra-striate visual areas develops during memory maintenance, but is absent in a control task matched in expectancy and difficulty to the memory task [62].

One could argue that the different duration of the experiments in laparoscopy compared with those performed with the robot represents a critical bias. However, such a criticism is ruled out both by the trend’s inversion as concerns inter-hemispheric coherence analysis and by the lack of any significant modification in coherence values comparing M1 and pre-SMA in different experimental conditions (laparoscopy vs. robotic surgery). In fact, our data seem to confirm the absence of anatomical connections between pre-SMA and the primary motor area [48]; as a consequence, non significant variations in mean values between the two experimental conditions could be likely due to the fact that these areas are tonically modulated by the same thalamic trigger.

### Inter-hemispheric coupling

Another intriguing result is about differences in inter-hemispheric connectivity; opposite from intra-hemispheric analysis, we highlighted higher coherence values in surgeons operating with the robot. We choose to analyze also variations between M1 and pre-SMA in order to completely isolate the contribution of interhemispheric pathways, since as previously mentioned these areas do not directly communicate within the same hemisphere, while a little cluster of fibers interconnect M1 and pre-SMA of the opposite side through corpus callosum [63].

In this scenario, coherence synchronization might be generated when callosal input, adding to the thalamocortical input, brings the depolarization of the callosally projecting neurons to a sufficient level of depolarization to trigger their intrinsic rhythm [46,64,65]; several studies have demonstrated that callosal axons selectively interconnect iso-orientation columns in humans implementing “collinearity”, a fundamental principle of Gestalt perception [66,67]. Long-range collinear facilitatory effects could serve as an important mechanism underlying the perception of continuity in visual patterns. For instance, tractography have revealed that bimanual object manipulation is mediated by callosal fibers that interconnect homologous areas and iso-oriented columns in the secondary somatosensory cortex [68]; a similar organization is present in visual cortex, too, thus preserving a retinotopic organization in primary as well as in associative visual areas [69]. Translating these observations into a behavioral point of view, one could speculate that robotic surgery is easier to learn for a surgeon, without necessarily requiring a long cognitive training neither a strong bio-sensitive feedback. Our results fit with data of Pellegrino and colleagues, who recently showed that in stroke patients, undergone to robot-aided rehabilitation program, interhemispheric connectivity between primary somatosensory areas got closer to a 'physiological level' in parallel with the acquisition of more accurate hand control [28,70].

This idea could be confirmed by analyzing power spectrum at different experimental sessions: in fact, the most represented frequencies in M1 in surgeons using the robot is the highest ones (p < 0.001), ranging from 12 to 30 Hz. It supports that upper alpha and beta rhythms may play an important role in long-range connectivity under different types of cognitive demands, whereas short-range coherence was not strictly associated with memory performance [71,72]. Moreover, this activity is known to originate mainly in the precentral motor cortex and is facilitated during execution as well as recognition of motor tasks [73]: thus, these results are in line with the existence of an action observation/execution matching network in the human brain, similar to that found in monkeys [74-76]. Although preliminary, our data could also suggest a preferential activation of human “mirror” system in surgeons operating with the robot compared with those trained in laparoscopy. However, minor contribution from premotor areas to the 15–20 Hz oscillations cannot be easily ruled out, although we used a high-density EEG device with 32 channels to improve the spatial selectivity of our recordings.

### Alternative explanations and possible pitfalls

A critical limitation of our study is about the impact of sub-cortical triggers on cortical activity; we cannot exclude the possibility that the significant differences in EEG coherence observed between the two experimental session is due, at least in part, to the activation of either cerebellar afferents or pathways from basal ganglia. A confounding contribution by cerebellum appears unlikely as cerebellar nuclei typically works at lower frequencies, in the range of theta band [77,78].

As regards delta band power, it might influence the overall results anyway, although not included in our analysis. However, it’s worth remembering that delta activity is a prominent feature of Non Rapid Eye Movement (NREM) sleep, mainly arising from cortical areas different from those we specifically evaluated, such as ventromedial prefrontal cortex, VMPFC [79-82]. Moreover, from a cognitive point of view, delta activity predominates when a functional disconnection of the cortex from environmental stimuli is required, for instance during mental calculation [83].

Another disadvantage is the *a priori* localization of pre-supplementary motor area: this region is better identified by electrocorticographic recordings, not obviously feasible in our study.

Finally, it is clear that functional connectivity (expressed in terms of coherence or temporal correlation) between different cortical areas depends on the type of experimental task used to estimate it. By changing the experimental task, functional coupling also changes. In our experiment, both the duration of motor tasks and the movements of surgeons during handling the different instruments were different. That could introduce spurious data in the interpretation of coherence analysis. However, as clearly demonstrated by Rupasov and colleagues [84,85], there is only a poor correlation between EEG signals and electromyographic (EMG) activity for several reasons: first, a large fraction of the EEG signal includes electrical activity unrelated to low-level motor variability; second, EMG signal cannot be described by a conventional model where the signal is normally distributed because it is composed by summation of many random sources; third, neural processing of cortically-derived signals by spinal circuitry may reduce the correlation between EEG and EMG signals.

Further research is required to confirm our preliminarily results; these observations could be helpful also in the evaluation of functional coupling in cortico-cortical connectivity in patients with robotic limbs or taking similar devices.

## Competing interests

The authors declare that they have no competing interests.

## Authors’ contributions

TB: conception and design of experiments, collection, analysis and interpretation of data, and writing of the paper. CM: collection, analysis and interpretation of data. ST: collection, analysis and interpretation of data. LB and MN: collection and analysis of data. LL, FM, MF and FS: design of experiments, writing of the article. All authors read and approved the final manuscript.
